# Structure-Guided Approach to Relieving Transcriptional Repression in Resistance to Thyroid Hormone *α*

**DOI:** 10.1128/MCB.00363-21

**Published:** 2021-12-06

**Authors:** Beatriz Romartinez-Alonso, Maura Agostini, Heulyn Jones, Jayde McLellan, D. Eilidh Sood, Nicholas Tomkinson, Federica Marelli, Ilaria Gentile, W. Edward Visser, Erik Schoenmakers, Louise Fairall, Martin Privalsky, Carla Moran, Luca Persani, Krishna Chatterjee, John W. R. Schwabe

**Affiliations:** a Leicester Institute of Structural and Chemical Biology, Department of Molecular and Cell Biology, University of Leicester, Leicester, United Kingdom; b Wellcome Trust-MRC Institute of Metabolic Science, University of Cambridge, Cambridge, United Kingdom; c Department of Pure and Applied Chemistry, University of Strathclyde, Glasgow, United Kingdom; d Laboratory of Endocrine and Metabolic Research, IRCCS Istituto Auxologico Italiano, Milan, Italy; e Department of Medical Biotechnology and Translational Medicine, University of Milan, Milan, Italy; f Erasmus Medical Center, Department of Internal Medicine, Academic Center for Thyroid Diseases, Rotterdam, The Netherlands

**Keywords:** resistance to thyroid hormone, thyroid hormone receptor *α*, transcriptional repression

## Abstract

Mutations in thyroid hormone receptor *α* (TR*α*), a ligand-inducible transcription factor, cause resistance to thyroid hormone *α* (RTH*α*). This disorder is characterized by tissue-specific hormone refractoriness and hypothyroidism due to the inhibition of target gene expression by mutant TR*α*-corepressor complexes. Using biophysical approaches, we show that RTH*α*-associated TR*α* mutants devoid of ligand-dependent transcription activation function unexpectedly retain the ability to bind thyroid hormone. Visualization of the ligand T3 within the crystal structure of a prototypic TR*α* mutant validates this notion. This finding prompted the synthesis of different thyroid hormone analogues, identifying a lead compound, ES08, which dissociates corepressor from mutant human TR*α* more efficaciously than T3. ES08 rescues developmental anomalies in a zebrafish model of RTH*α* and induces target gene expression in TR*α* mutation-containing cells from an RTH*α* patient more effectively than T3. Our observations provide proof of principle for developing synthetic ligands that can relieve transcriptional repression by the mutant TR*α*-corepressor complex for treatment of RTH*α*.

The physiological effects of thyroid hormones (TH; T4, thyroxine; T3, triiodothyronine) are mediated by their canonical action via nuclear thyroid hormone receptors (TRs; TR*α* and TR*β*) with differing tissue distributions, which regulate the transcription of target genes in a ligand-dependent manner (1). Unliganded TRs recruit a multiprotein complex which contains corepressor (CoR; e.g., nuclear receptor corepressor [NCoR] or silencing mediator of retinoic acid and thyroid hormone receptor [SMRT]) and histone deacetylase (HDAC) to inhibit target gene transcription. Receptor occupancy by a ligand (T3) promotes the dissociation of this corepressor complex with the relief of transcriptional repression and mediates the recruitment of a protein complex containing coactivators (e.g., NCoA-1/SRC-1, NCoA-2/GRIP-1, and NCoA-3/ACTR) with chromatin-remodeling activity, inducing the transcription of target genes (2). Short peptide sequences within these coregulators mediate interaction with the receptor: both NCoR and SMRT contain multiple receptor interaction domains (RIDs) encompassing isoleucine-rich amphipathic motifs (3–5). A leucine-rich (LXXLL) motif in coactivators mediates their binding to a liganded receptor, with conserved hydrophobic residues in the carboxyterminal alpha helix (helix 12) (Fig. 1B) of the TR being critical for this interaction (6).

Heterozygous, loss-of-function mutations in human TR*β* inhibit the function of their wild-type counterparts in a dominant negative manner via a mechanism involving the constitutive repression of target genes, causing resistance to thyroid hormone *β* (RTH *β*): this disorder is characterized by elevated circulating thyroid hormones (TH) and unsuppressed pituitary thyroid stimulating hormone (TSH) levels, consistent with hormone resistance and the predominant role of TR*β* within the pituitary-thyroid feedback axis (7). Conversely, RTH*α*, which is due to heterozygous TR*α* mutations, manifests with features of hypothyroidism (skeletal dysplasia, growth retardation, neurocognitive impairment, low metabolic rate, and reduced intestinal transit), reflecting hormone resistance in TR*α*-expressing tissues, but is paradoxically associated with near-normal levels of circulating TH. Nineteen different *THRA* defects have been recorded in 30 different families worldwide, with a significant subset (8/19) being frameshift or premature-stop mutations which disrupt the receptor carboxy terminus and are associated with a severe phenotype (8). These TR*α* mutants are transcriptionally inactive and fail to dissociate from the corepressor or recruit coactivator, mediating potent dominant negative inhibition of wild-type receptor function (9–11).

CRISPR/Cas9 editing of *Thra* has generated several mouse lines harboring different frameshift/premature stop mutations disrupting the TR*α* carboxy terminus, which exhibit a hypothyroid phenotype homologous to RTH*α*; significantly, the phenotype is less severe in animals harboring TR*α* mutants whose interaction with the corepressor is reduced (12). Crossing TR*α*-PV mice, another murine model of RTH*α* with a mutant TR*α* carboxy terminus (13), with mice expressing defective *Ncor1* which cannot be recruited to TR*α* (14), or treating TR*α*-PV mice with suberoylanilide hydroxyamic acid (SAHA), an HDAC inhibitor (15), ameliorates these phenotypes. Overall, these observations suggest that genetic or pharmacological disruption of the mutant TR*α*-CoR repression complex in RTH*α* might be beneficial.

Here, we have used biophysical (fluorescence anisotropy and circular dichroism) approaches to investigate the molecular properties of TR*α* mutants. Unexpectedly, we found that RTH*α*-associated TR*α* mutants could still bind T3. Validating this notion, we visualized T3 within the crystal structure of a prototypic TR*α* mutant, which was homologous to RTH*α*-associated mutant receptors. This finding prompted us to design, synthesize, and test TH analogues, identifying a lead compound which dissociated CoR from mutant human TR*α* more efficaciously than T3. This compound was more potent at preventing mutant human TR*α*-mediated developmental anomalies in a zebrafish model and induced greater TH target gene expression in patient-derived, TR*α* mutation-containing, primary cells studied *ex vivo*.

## Results

### Biophysical analyses of TR*α* mutant-coregulator interactions

**Fig 1:**
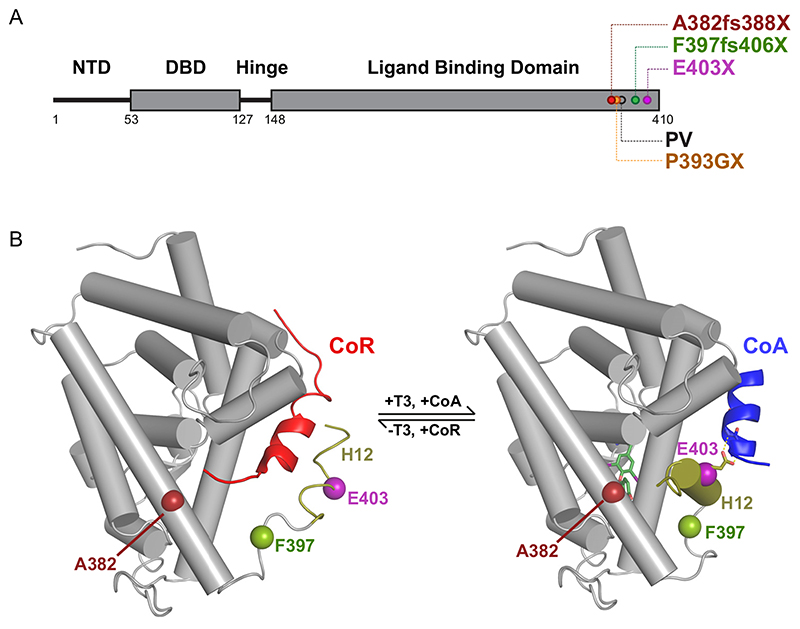
Location of human TR*α* mutations and WT TR*α* molecular switch. (A) Schematic representation of the full-length TR*α*, denoting the different functional domains of the receptor. Location of natural human mutations associated with RTH*α* (A382fs388X, F397fs406X, and E403X) and location of artificial mutation (P393GX) made for crystallization studies are indicated. (B) Structural model of TR*α* hormone-binding domain (PDB 2h79) in the unliganded state interacting with corepressor peptide (red) from the PPAR*α* LBD:SMRT complex (PDB 1kkq) (left panel), and in the liganded state bound to coactivator peptide (blue) from the PPAR*α* LBD:GRIP1 complex (PDB 1p54) (right panel). The position (A382, F397, E403) of natural human TR*α* mutations and the hydrogen bond between E403 and coactivator peptide are also shown.

We studied three human TR*α* mutants (A382fs388X, F397fs406X, and E403X) with disruption or truncation of the receptor carboxy terminus (Fig. 1A and B); these constitutively repress TH target genes, fail to activate their transcription in response to T3, and inhibit wild-type receptor action in a dominant negative manner (9, 11), suggesting that their interaction with coregulator proteins is altered. To determine whether these mutations mediated failure of corepressor displacement, failure of coactivator recruitment, or a combination of the two, we used a fluorescence anisotropy (FA) assay to investigate the ability of wild-type and mutant TR*α* ligand-binding domains (LBDs) to bind to peptide motifs derived from corepressor and coactivator receptor interaction domains in the presence or absence of T3.

**Fig 2:**
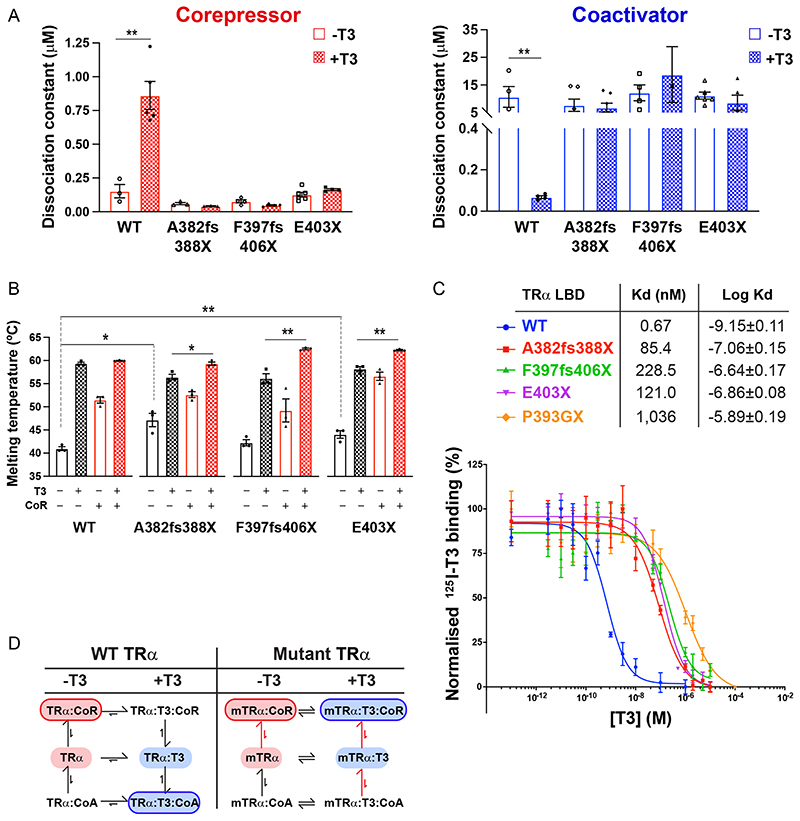
Interaction of human TR*α* mutants with corepressor and coactivator peptides (A) Binding affinities (dissociation constants) of wild-type and mutant TR*α* LBDs for corepressor in the absence (–T3) and presence (+T3) of T3 and for coactivator in the absence and presence of T3 in fluorescence anisotropy assays. Error bars indicate mean ± SEM (*n* = 5);**, P < 0.01. (B) Thermal stability (melting temperature) of wild-type and mutant TR*α* LBDs alone, in the presence of T3, with SMRT corepressor peptide, or with both SMRT and T3. Error bars indicate mean ± SEM (*n* = 5);*, P < 0.05; **, P < 0.01. (C) Radiolabeled T3 competitive binding assays of wild-type, mutant, and artificial mutant TR*α* showing the dissociation curves in the presence of increasing concentrations of unlabeled T3, and the dissociation constant (Kd) obtained. Data presented as mean ± SEM from two independent experiments performed in triplicate. (D) Scheme summarizing how the patient-derived mutations perturb the equilibria between ligand- and coregulator-bound species.

In the absence of T3, the interaction of both wild-type and mutant LBDs with the SMRT corepressor peptide is strong. Importantly, the mutant proteins A382fs388X and F397fs406X bind the corepressor with similar affinities to the unliganded wild-type receptor protein. In the absence of T3, neither the wild-type nor the mutant proteins exhibit detectable interaction with peptide from the GRIP1 coactivator (Fig. 2A).

As anticipated, the presence of saturating levels of T3 decreases the affinity of the wildtype TR*α* LBD for the SMRT corepressor peptide by almost 10-fold and increases the affinity for the coactivator peptide by several orders of magnitude (Fig. 2A). These changes in coregulator affinity underlie the ligand-induced molecular switch (Fig. 1B) which mediates the transition from repression to activation of target gene transcription by TR.

For the TR*α* mutants, we have delineated two molecular defects. First, their affinity for the corepressor in the presence of T3 is essentially unchanged; second, there is no T3-induced enhancement of coactivator interaction.

This behavior of TR*α* mutants as if the ligand were not present might suggest that the receptors are unable to bind to T3.

To test directly whether the mutant receptors were able to bind ligand, we used a circular dichroism (CD) assay to monitor the thermal stabilities of the wild-type and TR*α* mutant LBDs in the presence and absence of saturating levels of T3 and coregulator peptides (Fig. 2B). Interestingly, in the absence of ligand or coregulator peptides, the A382fs388X and E403X TR*α* mutants exhibited slightly greater thermal stability compared to the wild-type receptor. This suggests that helix 12 in unliganded, wildtype TR may confer a destabilizing effect which is not seen in TR*α* mutants lacking this carboxy terminal.

Strikingly, CD studies showed that all three TR*α* mutants were able to bind to the ligand (T3) and showed significant thermal stabilization, similar to that of the wild-type receptor. Similarly, in the absence of T3, both wild-type and mutant TR*α* proteins were significantly stabilized by corepressor binding. Significantly, for the three TR*α* mutants, the presence of both T3 and corepressor induced further stabilization of proteins, strongly suggesting that both the coregulator peptide and the ligand can bind simultaneously to the receptor LBD (Fig. 2B).

Given that all three TR*α* frameshift/truncation mutants lack helix 12, which caps the ligand binding pocket, with the A382fs388X mutant TR*α* also lacking a significant portion of helix 11, which forms one side of the ligand binding pocket, the finding that all mutant LBDs were able to bind T3 was unexpected.

Since the CD experiments were performed with saturating T3 concentrations, we determined the T3-binding affinity of the wild-type and mutant receptors in a competition assay using radiolabeled ligand (Fig. 2C). This confirmed that all three mutants bind T3 with sub-micromolar affinity, approximately 100-fold weaker than the wildtype receptor. Together, these studies suggest that the mutations in these patient-derived receptors exert their effects through an impairment in ligand- and coregulator-binding equilibria (Fig. 2D).

**Fig 3:**
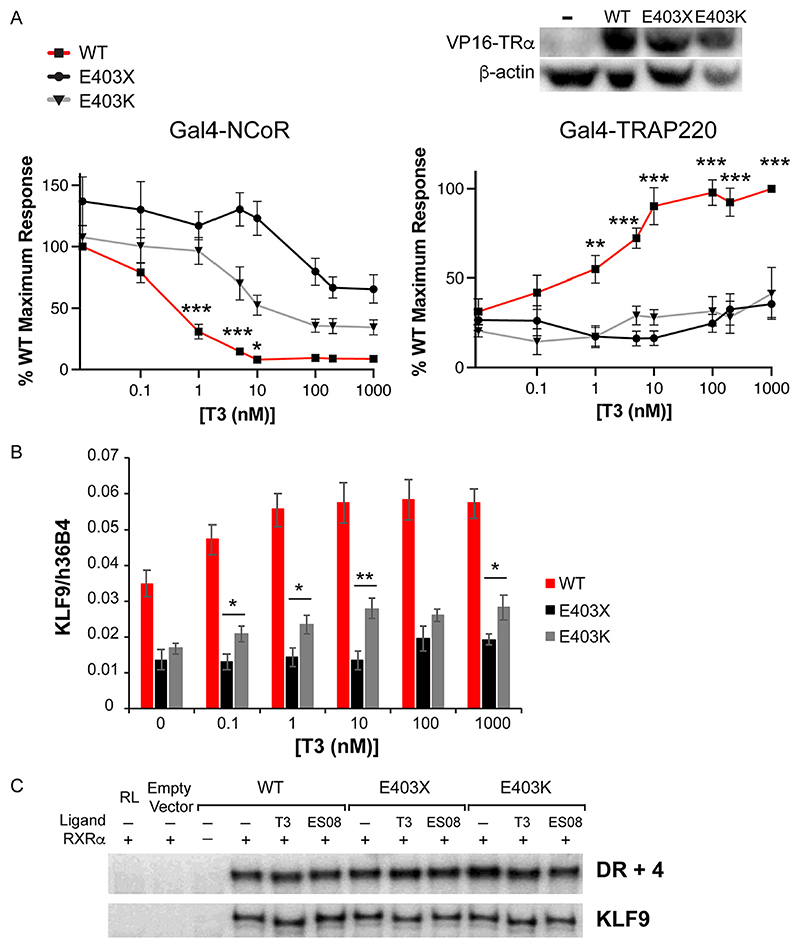
Comparison of the behavior of E403X and E403K mutant TR*α* receptors. (A) Two-hybrid assay to investigate the ability of ligand (T3) to dissociate NCoR and recruit TRAP220. *, P < 0.05; **, P < 0.01; ***, P < 0.001; ***, P < 0.0001 for comparisons of WT versus TR*α* mutants. (B) Analysis of KLF9 expression in patient-derived primary cells containing WT TR*α* or the E403X and E403K mutant TR*α* receptors. *, P < 0.05; **, P < 0.01 for comparisons of E403X versus E403K mutant TR*α*. (C) Electrophoretic mobility shift assays with WT, E403X or E403K mutant TR*α*, and RXR, with a canonical thyroid hormone response element (TRE) (DR 1 4) or a known TRE in the human KLF9 promoter, in the presence of 1 *μ*M T3 or 1*μ*M ES08.

To assess the relative contribution of defective corepressor release versus coactivator recruitment to the inhibition of TH action by mutant TR*α*, we analyzed patient-derived primary cells containing E403X TR*α*, a mutant which exhibits both defective corepressor dissociation and coactivator recruitment, versus patient-derived cells containing E403K TR*α*, a mutant with predominantly defective coactivator interaction (Fig. 3A). We observed that T3-dependent induction of KLF9, a TH target gene, was significantly more attenuated in E403X than in E403K mutant TR*α*-containing cells (Fig. 3B), supporting the concept that a receptor which cannot displace corepressor is more deleterious than one which simply cannot recruit coactivator. Importantly, electrophoretic mobility shift assays using either canonical (DR + 4) or natural (human KLF9 promoter) thyroid response elements showed that these differences in transcriptional activity were not due to the differential DNA binding properties of E403X or E403K TR*α* mutants in the presence of natural (T3) or synthetic ligands (ES08, see below) (Fig. 3C).

### Structure of a ligand-bound mutant TR*α*

**Fig 4:**
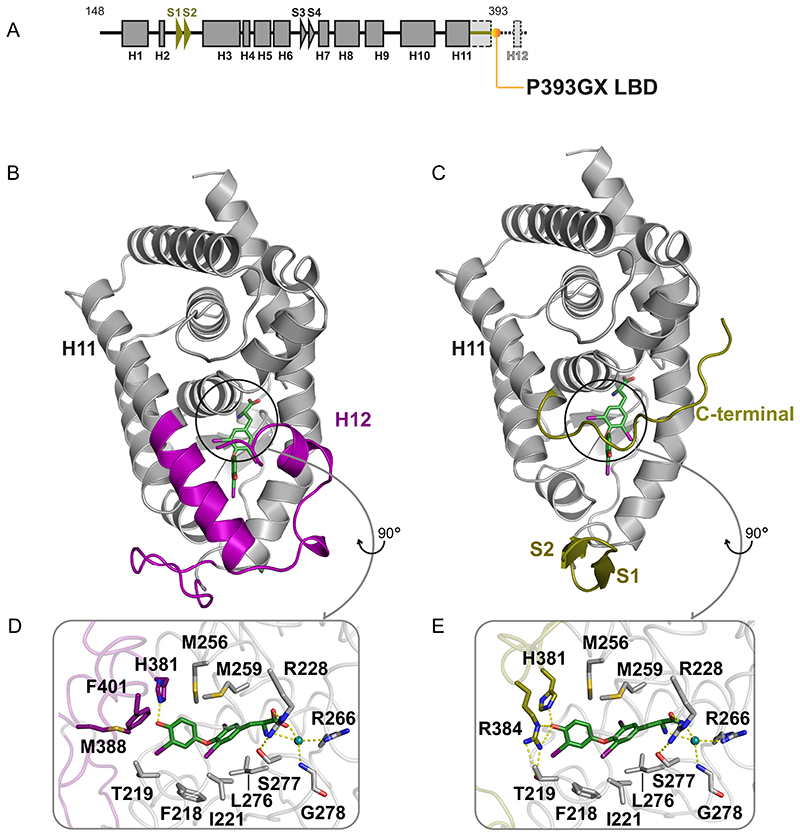
Comparison of wild-type and P393GX LBD structures. (A) Schematic representation of the P393GX LBD with alpha-helices (squares), beta-strands (arrowheads), and specific features of the P393GX mutant compared to the wild-type (highlighted in yellow). (B and C) Wild-type (B) and P393GX (C) LBD structures showing significantly different regions colored differently, with wild-type (purple) and P393GX (yellow). (D and E) Comparison of the wild-type (D) and P393GX (E) ligand-binding pockets, showing the different orientation of His 381 and different positions and interactions of Arg 384 residues. Key residues and helices are labeled with dashed lines denoting polar contacts.

To understand how the TR*α* mutants might bind ligand, we sought to crystallize the human mutant TR*α* LBDs in the presence of T3 and complexed with corepressor peptides, which likely reflects the dominant negative mutant receptor species in vivo. Unfortunately, we failed to obtain diffraction quality crystals, likely due to the presence of additional amino acids following frameshift mutations, which we would expect to be disordered. Accordingly, to model the human TR*α* mutants, we crystallized an artificial mutant receptor (P393GX) which was truncated at the carboxy terminus of helix 11 (Fig. 4A and C).

**Fig 5:**
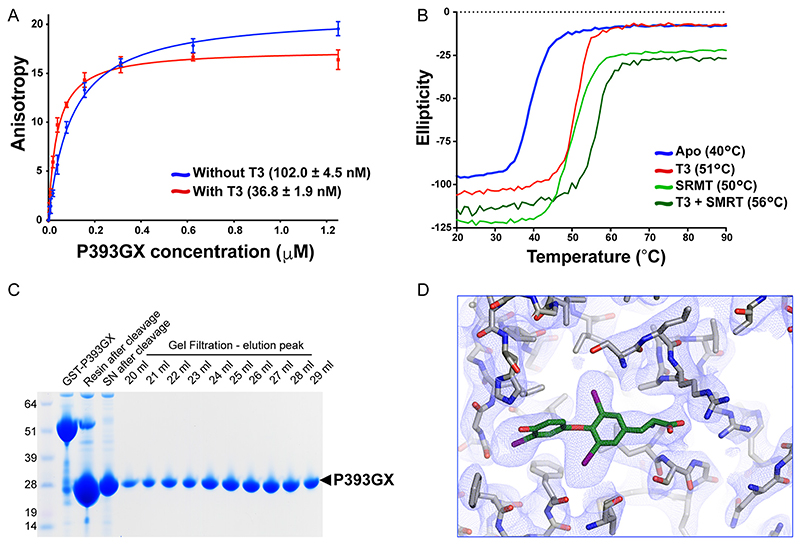
Characterization of P393GX LBD. (A) Corepressor binding assay of P393GX by fluorescence anisotropy showing saturating-binding curves and dissociation constant (Kd) of the P393GX binding to corepressor peptide in the absence and presence of T3. Error bars indicate mean ± SEM (n = 5). (B) Thermal stability (melting temperature) of P393GX in the apo form, with T3, with SMRT peptide, and with both T3 and SMRT peptide. (C) Purification of P393GX by GST and ion-exchange chromatography, illustrated via SDS-PAGE. (D) A 2F0-Fc electron density map contoured at 1.0 *σ* around the binding pocket of the protein, clearly showing T3 and the residues conforming the ligand binding pocket (structure deposited with PDB ID 7QDT).

Importantly, the P393GX TR*α* shows similar molecular behavior to the human TR*α* mutants. In fluorescence anisotropy assays, P393GX TR*α* interacts with corepressor peptides with comparable affinity to the human TR*α* mutant LBDs, with this interaction becoming stronger in the presence of T3 (Fig. 5A). As is the case with natural human TR*α* mutants, both ligand and corepressor peptide increase the thermal stability of P393GX TR*α*, with the greatest stabilization occurring in the presence of both T3 and SMRT-derived peptide, suggesting that P393GX LBD is able to bind T3 and SMRT corepressor simultaneously (Fig. 5B). Radiolabeled ligand binding studies confirmed T3 binding to P393GX mutant TR*α* with micromolar affinity (Fig. 2c), which was expected since the artificial mutant is more truncated than the natural human TR*α* mutants.

Small crystals of P393GX in complex with T3 were obtained which diffracted to 3.0 Å. The structure contains one P393GX molecule (amino acids 156 to 393) and one T3 molecule in the asymmetric unit (Fig. 5C; Table 1). Much of the overall organization of the LBD is essentially the same as that of the wild-type receptor protein. However, despite having the potential to form a complete helix 11 (aa 363 to 393), in the P393GX mutant TR*α* this helix terminates at Ala 379, with the peptide backbone turning at this point to form an extended coil which runs antiparallel to the adjacent helix 5 toward the corepressor-binding site.

The T3 ligand could be placed unambiguously in the hydrophobic cavity of P393GX TR*α* LBD (Fig. 5D) and binds very similarly to its occupancy of wild-type receptor LBD (Fig. 4D and E). However, T3 interactions with Met 388 and Phe 401 are lost and the orientation of His 381 is altered. Arg 384 makes a new interaction with the ligand in the P393GX LBD, donating a hydrogen bond to the 49 hydroxyl of the outer ring of T3. Interestingly, the lack of the carboxy-terminal end of helix 11 and the joining of the loop to helix 12 creates space for residues amino-terminal to helix 3 to form a short anti-parallel *β*-sheet, which is not present in the wild-type receptor.

Remarkably, given that so much of the receptor carboxy terminus is missing, the ligand binding cavity in P393GX mutant TR*α* is almost completely enclosed due to the rearrangement of the carboxy-terminal portion of helix 11. Indeed, the volume of the ligand-binding cavity in P393GX is only slightly greater than that of the wild-type receptor (≈200 Å^3^ versus ≈160 Å^3^, as calculated using POCASA [16]). There is a small opening to the surface of the receptor around the 49 hydroxyl and iodine of the outer ring of T3, suggesting that the mutant receptor could likely accommodate a larger ligand in these positions. Since P393GX mutant TR*α* is capable of binding T3 and corepressor (Fig. 5B), we surmise that if the corepressor peptide binds the receptor at the expected site, part of the rearranged helix 11 would likely be displaced to accommodate this interaction.

### Novel thyroid hormone analogues displace corepressor from human TR*α* mutants

**Fig 6:**
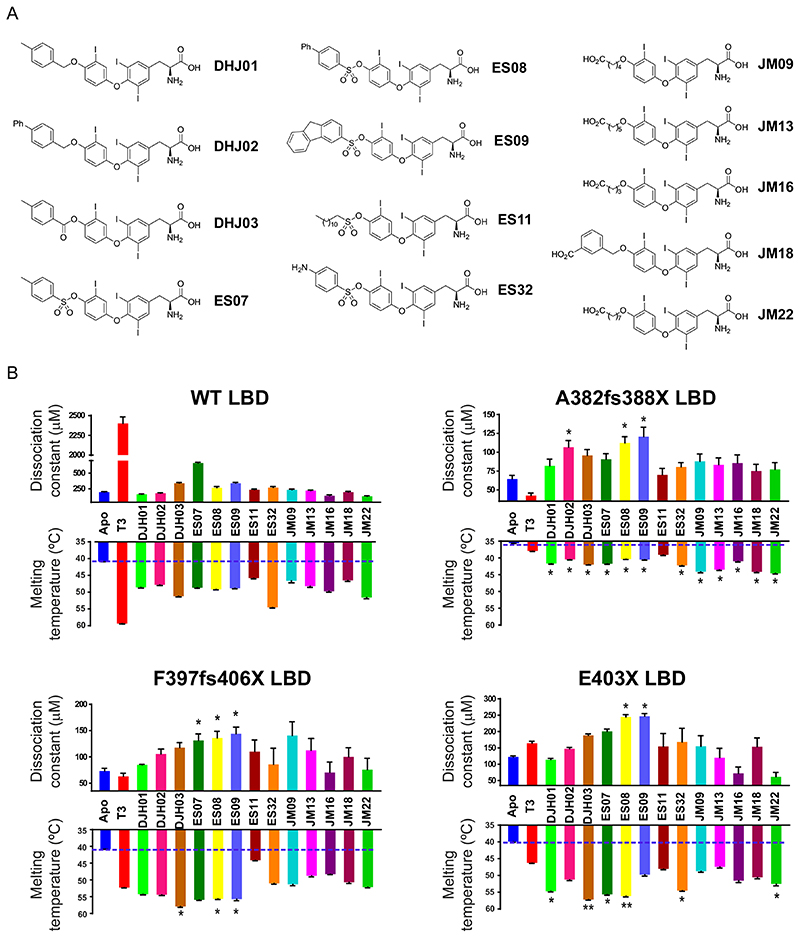
Effect of T3 and analogues on binding of wild-type and mutant TR*α* LBDs to corepressor. (A) Chemical structures of T3 analogues. Three series of novel T3 derivatives are shown. Each series contains an extension at the 4′ hydroxyl group of T3 linked with either an ether, ester, or sulfonate ester functional group. The extensions differ in size, hydrophobicity, and conformational flexibility. The JM ligands also have a carboxylic acid at the distal end of the extension. (B) Corepressor-binding affinity (dissociation constants) in fluorescence anisotropy assays (upper graphs) and thermal stability (melting temperature; bottom graphs) of wild-type and mutant TR*α* LBDs in the presence of T3 and different analogues. Anisotropy values were measured at each concentration of the protein:T3 analogue (molar ratio, 1:2) mixture to generate saturation-binding curves, from which the equilibrium dissociation constant of the interaction (Kd) was calculated. Error bars indicate ± SEM (n = 3); *P < 0.05 for comparisons of TR*α* mutants and T3 versus TH analogues. Melting temperatures of apo receptor proteins (blue) with either T3 (red) or T3 analogues (different colors), obtained by measuring changes in ellipticity of samples at 222 nm over a temperature range (20 to 90°C, 1-degree steps), are also shown. Error bars indicate mean 6 SEM. (n = 3); *, P < 0.05; **, P < 0.01 for comparisons of TR*α* mutants and T3 versus TH analogues.

The finding that both natural and artificial TR*α* mutant LBDs retain the ability to bind T3 raised the possibility of designing novel ligands that are better able to displace corepressor, thereby alleviating transcriptional repression by mutant human TR*α*. We synthesized a series of ligands in which the hydroxyl group of T3 was modified with either ether, ester, or sulfonate ester linkages of varying size, hydrophobicity, and flexibility (Fig. 6A).

Novel ligands were tested at saturating concentrations in fluorescence anisotropy and thermal stability assays using wild-type and human TR*α* mutant LBDs. Compared to the unliganded proteins, all compounds increased the thermal stability of wild-type and mutant LBDs, indicating their ability to bind these proteins. Strikingly, whereas T3 stabilized the wild-type TR*α* LBD better than the synthetic analogues, several of the novel ligands (including ES08) stabilized mutant TR*α* proteins more effectively than T3; this suggests that the additional groups in these modified ligands enabled them to better occupy the aberrant ligand-binding pockets of the mutant proteins (Fig. 6B, lower panels).

**Fig 7:**
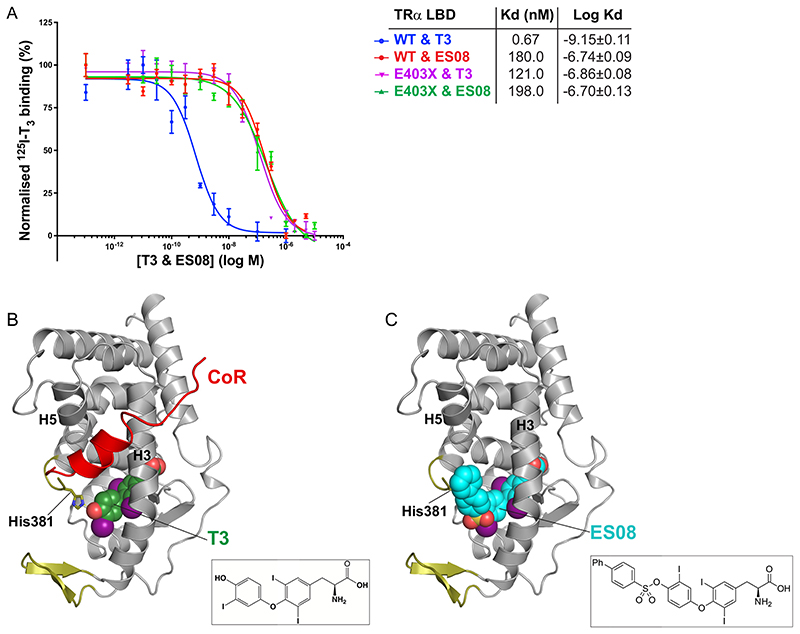
Binding affinity and fitting of ES08. (A) Competitive binding assay with radiolabeled T3, wild-type or mutant TR*α*, and increasing concentrations of unlabeled ES08, indicating dissociation constants (Kd) obtained. Data shown are the mean ± SEM from two independent experiments performed in triplicate. Note that the data for WT binding T3 is included for comparison, duplicated from Fig. 2C. (B) Model of corepressor binding to P393GX:T3 complex. T3 is shown in green with the SMRT corepressor peptide in red. Interaction of the LBD and corepressor peptides is mediated by corepressor recruitment in a hydrophobic groove formed by H3 and H5 of the LBD. Due to the lack of H12 in TR*α* mutants, there is no disruption of the CoR binding surface, and the corepressor remains interacting with the mutant receptors even in the presence of T3 forming the ternary complex. (C) Model of ES08 (cyan) binding to P393GX. Here, in contrast to T3, the extension at the 4′-hydroxyl of the outer thyronine ring potentially disrupts the CoR binding surface of the LBD and, consequently, ES08 interferes with corepressor interaction.

Fluorescence anisotropy assays measured the ability of ligands to perturb the binding of wild-type and human TR*α* mutant LBDs to corepressor peptide (Fig. 6B, upper panels). Many synthetic ligands promoted the dissociation of corepressor from wild-type TR*α*, but they did so less effectively than T3, presumably due to their inability to rearrange helix 12 into its active, corepressor-displacing conformation (17). However, two compounds, ES08 and ES09, mediated dissociation of corepressor from human TR*α* mutants more effectively than T3, particularly in the case of the mutant receptor E403X (Fig. 6B, upper panels). Competition assays with radiolabeled T3 confirmed that both wild-type and E403X TR*α* were able to bind ES08 with submicromolar affinity. For wild-type TR*α*, ES08 binding is much weaker than T3. However, importantly, the E403X mutant TR*α* binds T3 and ES08 with similar affinities (Fig. 7A).

### Efficacy of TH analogues in mediating TR*α*-corepressor dissociation in cells

To compare the efficacy of T3 and synthetic ligands in promoting corepressor dissociation from TR*α* in cells, we tested them in cellular two-hybrid protein-protein interaction assays with co-expressed VP16-full length TR*α* and Gal4-corepressor fusions.

**Fig 8:**
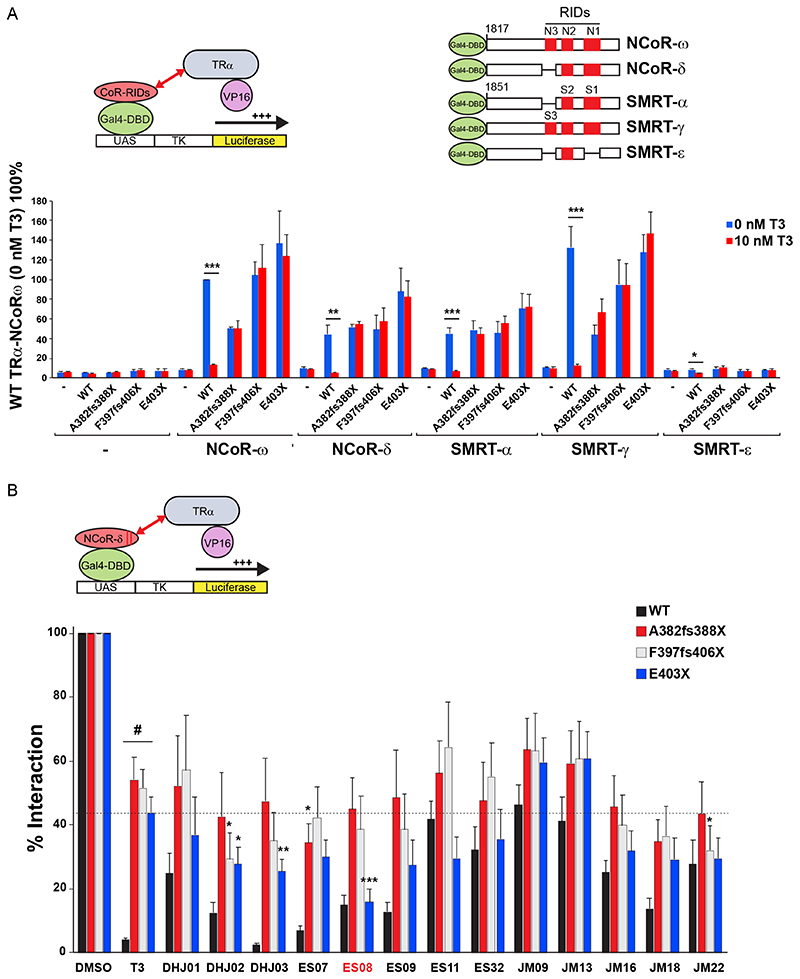
Interaction of VP16-TR*α* with GAL4-corepressor in mammalian cells. (A) Schematic representation of the cellular two-hybrid assay (left) and different human GAL4-corepressor isoforms (with locations of nuclear receptor interaction domains in red) tested (right). Below, this interaction of WT or mutant VP16-TR*α* fusions with different corepressor isoforms in the absence or presence of T3 is shown. Results are presented as mean 6 SEM for at least 5 independent experiments performed in triplicate. *, P < 0.05; **, P < 0.01; ***, P < 0.001 for 0 nM T3 versus 10 nM T3 comparisons. (B) Interaction of GAL4-NCOR-*δ* with VP16-WT or mutant TR*α* fusions in the presence of vehicle (DMSO), 100 nM T3, or a different analogue. Results from at least 5 independent experiments are shown as percentages of interaction for WT or TR*α* mutants with NCoR-*δ* with vehicle. *, P < 0.05; **, P < 0.01; ***, P < 0.001 for comparisons of TR*α* mutants and T3 versus TR*α* mutants and analogue; #P < 0.001 for comparison of WT versus TR*α* mutants with 100-nM T3.

Both unliganded wild-type and mutant VP16-TR*α* fusion proteins exhibited strong interaction with Gal4-CoR fusions containing different isoforms of human NCoR (NCoR-*ω* and NCoR-*δ*) or SMRT (SMRT-*α* and SMRT-*γ*) (18). T3 exposure readily dissociated all CoR isoform fusions from wild-type receptor but not from human TR*α* mutants. SMRT-*ε*, a corepressor isoform lacking two receptor interaction motifs (S1 and S3) did not interact with either wild-type or mutant TRs (Fig. 8A).

**Fig 9:**
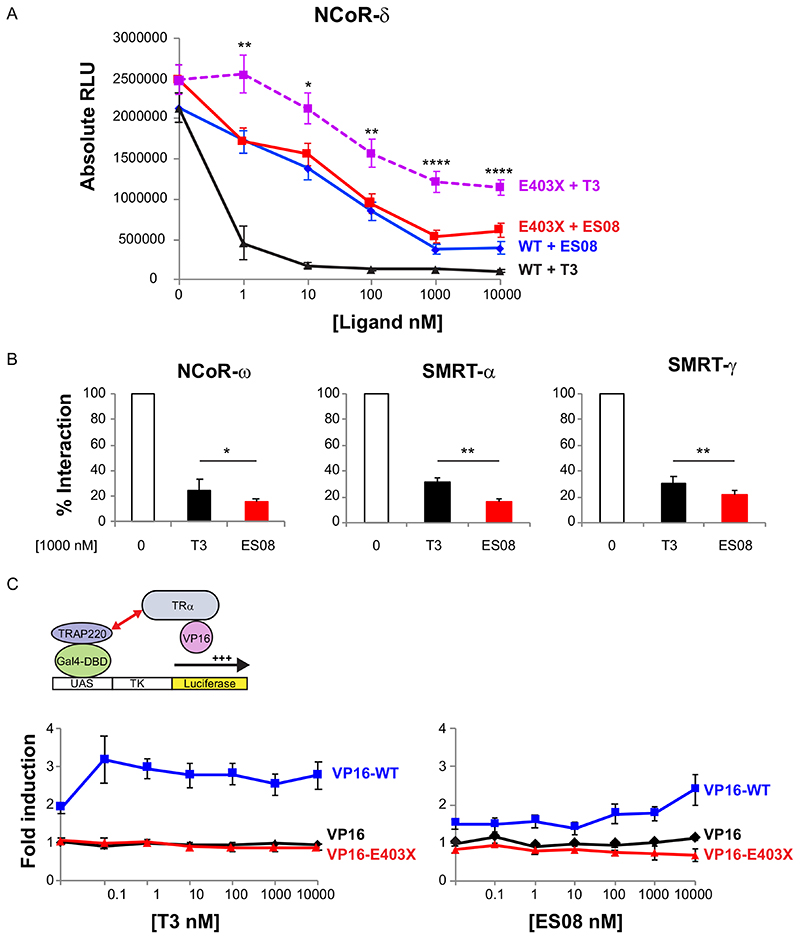
ES08 induces greater dissociation of corepressors from E403X mutant TR*α* than T3 does, but fails to recruit Gal4-TRAP220 coactivator in cellular protein interaction assays. (A) Dissociation of VP16-WT or VP16-E403X mutant TR*α* from GAL4-NCOR-*δ* with increasing concentrations (0 to 10,000 nM) of T3 or ES08. Results, shown as relative luciferase units (RLU) normalized to *β*-galactosidase activity, are given as the mean ± SEM of at least 5 independent experiments performed in triplicate. *, P < 0.05; **, P < 0.01; ***, P < 0.001; ****, P < 0.0001 for comparison of E403X plus T3 versus E403X plus ES08. (B) Interaction of VP16-E403X mutant TR*α* with different Gal4-corepressor isoform fusions in the absence of ligand (0), 1,000 nM T3, or ES08. Results from at least 5 independent experiments performed in triplicate are expressed as a percentage of WT TR*α*-corepressor interaction in the absence of T3. *, P < 0.05; **, P < 0.01 for comparison of E403X plus T3 versus E403X plus ES08. (C) Inability of VP16-E403X mutant TR*α* (red line) to recruit GAL4-TRAP220 coactivator fusion over a range (0 to 10,000 nM) of T3 or ES08 concentrations. The data are expressed as fold induction relative to cells transfected with Gal4-TRAP220 and VP16 alone and are shown as the mean ± SEM of at least 5 independent experiments each performed in triplicate.

Next, we compared the relative efficacy of T3 versus different TH analogues (each at 100 nM) in promoting corepressor dissociation from wild-type or mutant TR*α* using two-hybrid assays. Compared to the complete or partial dissociation of corepressor from wild-type receptor or TR*α* mutants with T3 exposure, most TH analogues exhibited comparable or inferior efficacy (Fig. 8B). Notably, when tested with a particular human mutant receptor, E403X TR*α*, a single compound, ES08, promoted greater dissociation of corepressor from mutant TR*α* than T3 did (Fig. 8B, highlighted). We confirmed that ES08 promotes greater corepressor dissociation from E403X mutant TR*α* than T3 does when tested over a range of ligand concentrations (Fig. 9A). Furthermore, compared to T3, ES08 promotes greater dissociation of other corepressor isoforms (NCoR-*ω*, SMRT-*α*, and SMRT-*γ*) from E403X mutant TR*α* (Fig. 9B). However, neither ES08 nor T3 was able to overcome the inability of E403X mutant TR*α* to recruit coactivator (Fig. 9C).

### Efficacy of TH analogue in E403X mutant TRa-expressing zebrafish and patient-derived primary cells

**Fig 10:**
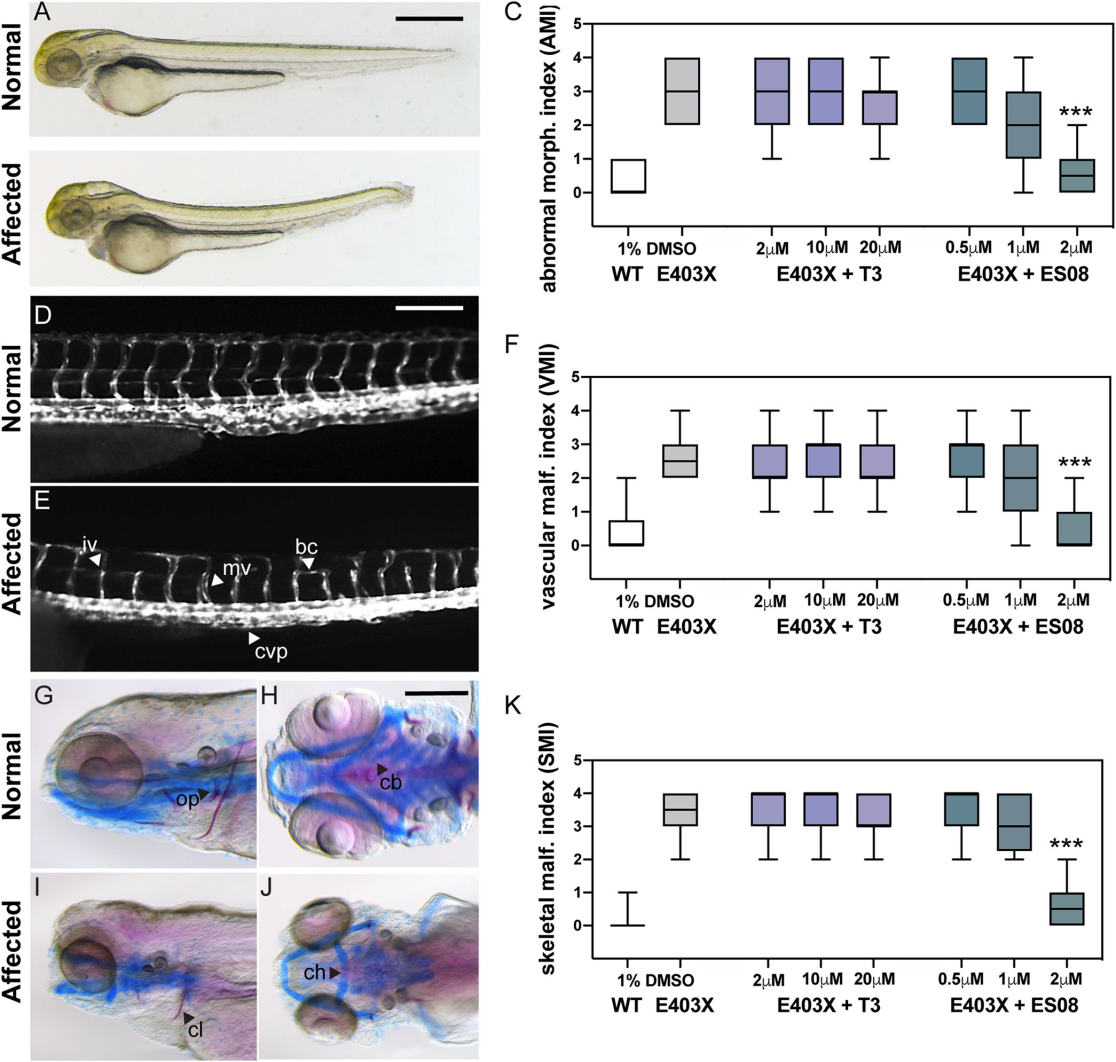
Effect of T3 and ES08 on E403X mutant TR*α*-expressing zebrafish embryos and E403X TR*α* mutation-containing, patient-derived inducible pluripotent stem cells. (A and B) Normal or abnormal morphology of embryos injected with hTR*α* WT or hTR*α* E403X mRNAs at 3 days postfertilization (dpf). Cerebral edema (ce), pericardial edema (pe), altered body curvature (bc), and thickening of caudal vein plexus (cvp) were used to calculate an abnormal morphology index (AMI, 0 = unaffected, 1 to 4 = affected). Scale bar = 250 *μ*m. Boxplots of AMI for WT (open) or E403X injected zygotes treated with DMSO (gray), T3 (purple), or ES08 (gray-green) are shown in panel C. (D and E) transgenic tg(kdrl:EGFP) line was used to visualize normal or disrupted vascular architecture of the trunk-tail region of embryos at 4 dpf (lateral view, head to the left). Anomalies, including incomplete vessel (iv), aberrant branching (ab), misplaced vessel (mv), and reduced caudal vein plexus (cvp) area, shown in panel E, were used to calculate a vascular malformation index (VMI 0 = unaffected, 1 to 4 = affected). Scale bar = 50 *μ*m. Boxplots of VMI of WT and E403X injected zygotes treated with DMSO (gray), T3 (purple), or ES08 (gray-green) are shown in panel F. (G to J) Normal or defective cranial cartilage development and bone mineralization in 5 dpf embryos stained with Alcian Blue (in blue) and Alizarine (in red), respectively. Absent or reduced mineralization of cleitrum (cl) and operculum (op) (visible in lateral view), and malformations of ceratohyal (ch) and the five ceratobranchial (cb) arches of cartilages (visible in ventral view) were quantified to compute a skeletal malformation index (SMI). Scale bar = 100 *μ*m. (K) Box plots of SMI of WT and E403X mutant TR*α*-expressing embryos following exposure to DMSO, T3, or ES08. Indices shown are mean ± SEM for pools of 60 embryos per condition, from at least 4 independent experiments. P < 0.001 (***) for comparison of malformation indices in E403X TR*α*-expressing embryos after exposure to DMSO versus exposure to ES08.

We evaluated the efficacy of ES08 versus T3 in a zebrafish model of RTH*α*, as described previously (19). Following microinjection of zygotes with either wild-type receptor or E403X mutant TR*α* mRNAs, the embryos derived from E403X TR*α* mutant-injected zygotes exhibited multiple developmental anomalies, consisting of abnormal indices of morphology (AMI), vascular malformation (VMI) or skeletal malformation (SMI), whereas embryos from wild-type TR*α*-injected zygotes were unaffected (Fig. 10).

**Fig 11:**
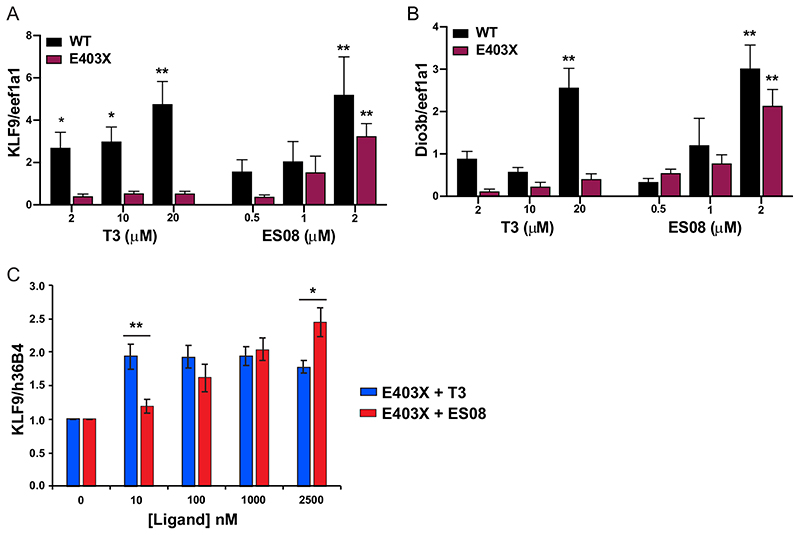
In vivo analysis of TR*α* target genes. (A and B) Expression of thyroid hormone receptor target genes (KLF9 and Dio3b) in WT and E403X mutant TR*α*-injected zygotes exposed to vehicle (DMSO), T3, or ES08. Results are expressed as fold changes versus DMSO-treated cells. *, P < 0.05; **, P < 0.01 for comparison with vehicle-treated embryos. (C) Expression KLF9 (a thyroid hormone receptor target gene) in inducible pluripotent stem cells derived from patient harboring an E403X TR*α* mutation, with increasing concentrations of T3 or ES08 analogue. Results are expressed as fold changes versus nontreated cells: *, P < 0.05; **, P < 0.01 for comparison of E403X with T3 versus E403X with ES08.

Despite exposure to T3 at high concentrations (2 to 20 *μ*M), these developmental anomalies persisted in embryos derived from E403X TR*α*-injected zygotes, whereas their exposure to ESO8 (2 *μ*M) prevented morphological, vascular, and skeletal malformations (Fig. 10C, F, and K). Furthermore, in E403X mutant TR*α*-expressing embryos, lower concentrations of ES08 (2 *μ*M) induced greater expression of known thyroid hormone-responsive target genes than did high concentrations (2 to 20*μ*M) of T3 (KLF9 [Fig. 11A]; Dio3b [Fig. 11B]).

Finally, we compared the properties of ES08 versus T3 when incubated with primary, patient-derived, E403X mutant TR*α*-containing, inducible pluripotent stem cells. Compared to T3, ES08 at a higher concentration (2.5 *μ*M) induced greater expression of KLF9, a known thyroid hormone-responsive target gene (Fig. 11C).

## Discussion

Severe RTH*α* is caused by a molecular mechanism involving the constitutive interaction of mutant TR*α* with corepressor, failure of corepressor release, and failure of coactivator recruitment in a ligand-dependent manner, resulting in silencing of gene transcription. Unexpectedly, given unmeasurable radiolabeled T3-binding to mutant TR*α* in previous assays (9), our biophysical studies together with radiolabeled T3-binding assays showed that these human TR*α* mutants retain hormone-binding ability. This was confirmed by visualizing T3 within the crystal structure of an artificial TR*α* mutant, prototypic of receptor mutants that commonly cause RTH*α*. This finding prompted the synthesis and evaluation of TH analogues, leading to the successful identification of a single compound which dissociates corepressor from human mutant TR*α* more effectively than T3 does. This synthetic ligand prevented phenotypic abnormalities in a zebrafish model of RTH*α*, and induced target gene expression in TR*α* mutation-containing cells from an RTH*α* patient, more efficaciously than T3.

The crystal structure of the P393GX TR*α* LBD reveals that the majority of the protein-ligand interactions are preserved in the mutant receptor despite the loss of much of H11 and H12. This accounts for why the mutant receptors retain the ability to bind thyroid hormone, albeit with substantially reduced affinity. The position of the car-boxy-terminal portion of H11 in the structure appears to occlude the CoR binding region. This would appear to be an artifact of crystallization since we have shown experimentally that, like the natural TR*α* mutants, the P393GX receptor binds CoR bothin the presence and in the absence of T3. We would predict that this region will not adopt a single conformation in solution.

Almost half (8/19) of the mutations causing RTH*α* which have been described hitherto are associated with a severe hypothyroid phenotype and disrupt the receptor carboxy terminus in a manner analogous to the TR*α* mutants we have studied here (9, 11, 20–23). To date, thyroxine treatment of such patients has been associated with a poor (10) or partial (11) clinical response. This may be because thyroxine therapy at conventional dosages does not lead to corepressor dissociation. New ligands that maximize the likelihood of corepressor dissociation from mutant TR*α* may be a more successful treatment.

Incomplete ligand-dependent release of corepressor from mutant TR*α* despite its ability to bind T3, therefore, provided the rationale for synthesizing TH analogues, identifying a compound, ES08, which mediated greater corepressor displacement from the E403X mutant. ES08 and ES09 are sulfonate ester T3 analogues bearing a biaryl or 9H-fluorene substituent, respectively. In the structure of ES08, it is possible that the change in geometry induced by the sulfonate ester link, together with the relative rigidity of the extension, sterically disrupts corepressor binding to the cleft on the TR LBD surface, thereby lowering its affinity for corepressor binding (Fig. 7B and C). We speculate that the presence of part of helix 12 in E403X mutant TR*α* accounts for the ability of this analogue to displace corepressor more effectively from this particular TR*α* mutant.

One limitation of our study was the replacement of the phenol group of T3 with a sulfonate ester in ES08, disrupting the known interaction of its hydroxyl group with Histidine 381 in TR, which may have compromised its receptor-binding affinity. Accordingly, in the future, we will synthesize and test different ligands, restoring this phenol moiety and modifying T3 in the 5′ position instead, since structural modeling suggests that such analogues would be better orientated to displace corepressor. Furthermore, although the synthetic analogue we have identified, ES08, was only effective with a specificTR*α* mutant, our studies suggest that it may be possible to generate ligands that can better displace corepressor from this general subclass of mutant receptor. As the comparison of the E403X and E403K mutants suggests, corepressor displacement is beneficial even in the absence of coactivator recruitment. It is striking that corepressor displacement, at high ES08 concentrations, has a significant beneficial effect in the zebrafish model. The recent recognition that a thyroid hormone analogue (triiodothyroacetic acid) which is used to treat resistance to thyroid hormone *β* acts via this mechanism (24) provides added justification for this approach.

Other approaches to relieving transcriptional repression by the mutant TR*α*-core-pressor complex in RTH*α* have limitations. In a recent study, abrogating NCoR-TR interaction in TR*α*-PV transgenic mice did not reverse skeletal abnormalities, suggesting that transcriptional repression by mutant TR*α* complexed with other corepressors (e.g., SMRT) may mediate this phenotype (25). Similarly, targeting the TR*α*-corepressor repression complex pharmacologically with an HDAC inhibitor may not be effective if transcriptional repression operates via diverse complexes containing different HDACs which may not be sensitive to such inhibition (15). Histone deacetylases are also components of complexes with other nuclear receptors and transcription factors (26, 27), and nonspecific inhibition of histone deacetylase enzyme activity may derepress these pathways, causing off-target effects.

Overall, our observations provide a proof of concept for the synthesis of designer ligands, targeting aberrant mutant TR*α*-corepressor interaction, to alleviate receptor dysfunction in resistance to thyroid hormone *α*.

## Materials And Methods

### Expression and purification of TR*α* LBDs

The human WT, A382fs388X, F397fs406X, E403X, and P393GX LBDs (residues 148 to 410, 148 to 387, 148 to 402, and 151 to 393) were cloned into a pGEX2T (GE Healthcare) vector containing an amino-terminal glutathione S-transferase (GST) purification tag followed by a TEV protease cleavage site. TR*α* LBDs were expressed in E. coli Rosetta (DE3) (Novagen) by growing the transformed Rosetta (DE3) at 37°C in 2xTY until A_595_ = 0.1, then inducing with 40 *μ*M isopro-pyl-D-1-thiogalactopyranoside (IPTG) and growing overnight at 20°C. The bacterial cells were lysed by sonication in a buffer containing 1 × phosphate-buffered saline (PBS), 1 mM dithiothreitol (DTT) and complete EDTA-free protease inhibitor (Roche). The soluble protein was bound to glutathioneSepharose (GE Healthcare), and washed with a buffer containing 1 × PBS, 0.5% Triton X-100, and 1 mM DTT. Next, the bound protein was washed with TEV cleavage buffer containing 20 mM Tris-HCl (pH 8), 100 mM NaCl, and 1 mM DTT. The GST tag was removed by incubation with TEV protease (100:1 molar ratio) overnight at 4°C.

Eluted proteins were loaded onto 5-mL HiTrap Q HP ion exchange columns (HiTrap Q HP IEX), previously equilibrated in low-salt buffer (20 mM Tris-HCl [pH 7.4], 50 mM NaCl, 1 mM DTT). The protein was eluted with a 50 to 500 nM NaCl gradient at a flow of 1.5 mL/min. Partially purified samples were further purified on a Superdex 75 10/300 gel filtration column (GE Healthcare Bio-Science) in 30 mM Tris-HCl (pH 8.0), 50 mM NaCl, 5% [vol/vol] glycerol, 1 mM DTT, and 0.5 mM EDTA. Pooled fractions were buffer exchanged and concentrated in 1 × PBS, 1 mM DTT buffer for fluorescence anisotropy and circular dichroism assays, and in 20 mM Tris (pH 8.0), 50 mM NaCl, and 1 mM DTT for crystallization studies.

### Radiolabeled T3-binding assays

Homologous competitive binding assays were performed using triiodothyronine (T3) labeled with ^125^I (^125^I-T3, PerkinElmer). Recombinant WT, mutant, and artificial mutant TR*α* LBDs were incubated with 0.02 nM ^125^I-T3 in binding buffer (20 mM Tris [pH 8], 50 mM KCl, 1 mM MgCl_2_, 10% glycerol, 5 mM DTT) in the presence of increasing amounts of unlabeled competing T3 (0 to 100 *μ*M). Appropriate protein concentrations were determined experimentally to give 10% of the total radioactivity of the assay, securing a good signal (total binding) to noise (nonspecific binding) ratio and preventing the ligand depletion effect. Following 1 h of incubation at 37°C, bound T3 was separated from unbound T3 by passage through a filter membrane (Millipore HA Filters, 0.45 *μ*m) under vacuum, followed by three washes with 2 mL of ice-cold binding buffer. Filters containing TR-bound ^125^I-T3 were measured in a *γ*-counter.

A competitive binding assay was performed following the same procedure in the presence of increasing amounts of unlabeled competing ES08 (0 to 10 *μ*M) using recombinant WT and E403X TR*α* LBDs.

A half-maximal inhibitory concentration (IC_50_), which indicates the amount of ligand that causes 50% inhibition of radioligand binding, was determined by plotting the radioactivity values obtained at every cold-competing T3 concentration, using the GraphPad Prism and a nonlinear regression analysis. Since the dissociation constant, Kd, and the inhibitor constant, *K_i_*, should be the same as the radioactive ligand and the competing ligand are the same, for this type of experiments the Kd of the binding is calculated by subtracting the concentration of radioligand from the IC_50_ value obtained from the curve, as in the following equation: Kd = *K_i_* =IC_50_ – [radioligand conc.].

### Fluorescence anisotropy and circular dichroism

Two peptides were designed for use in the fluorescence anisotropy assay: an N-terminal, FITC-labeled, 14-aa-length peptide with a sequence based on the interaction domain 1 of the SMRT corepressor protein (RID1 residues 2346 to 2360: Ac-STNMGLEAIIRKALMG-NH_2_), containing the corepressor NR recognition motif LxxxIxxx[I/L]; and a C-terminal, BODIPY-TMR-labeled, 16-aa-length peptide with a sequence based on the second NR interaction box of the GRIP1 coactivator protein (NID2 residues 686 to 700: Ac-KHKILHRLLQDSSC-NH_2_), containing the coactivator NR recognition motif LxxLL.

Fluorescence anisotropy (FA) experiments were performed in black 96-well assay plates (Corning Life Sciences). Multiple titrations were performed using fixed concentrations of SMRT and GRIP1 peptides (5 nM) with increasing concentrations of TR*α* LBDs (0 to 5*μ*m) in a final volume of 50 *μ* L of assay buffer (1 × PBS, 0.01% [vol/vol] Triton X-100, 0.1 mg/mL bovine serum albumin [BSA]). For the assays in the presence of T3 or T3 analogues, increasing concentrations of the mixture protein:T3 or protein:T3 analogues in a 1:2 molar ratio were used. After incubation at room temperature for 5 min with slow shaking and centrifugation of the plates, the FA value was measured at each receptor concentration in a VICTOR X5 multilabel plate reader (Perkin Elmer, Singapore), using a 480-nm excitation filter and 535-nm emission filters to measure FITC emission, and a 542-nm excitation filter and 572-nm emission filters to measure BODIPY fluorescence. Blank fluorescence values were subtracted in each polarization plane. FA values obtained at each protein concentration were used to generate saturation-binding curves that were subsequently used to calculate the equilibrium dissociation constant of the interaction (Kd), using Prism software (GraphPad) and nonlinear regression analysis.

Thermal unfolding of proteins was monitored by CD spectroscopy over a wavelength range of 200 to 250 nm, using a Chirascan Spectrometer (Applied Photophysics) equipped with a temperature controller (Quantum Northwest TC125). CD spectra were measured from samples in 1-mm-path-length quartz cuvettes, using a scanning speed of 100 nm/min, a spectral bandwidth of 1 nm, and a response time of 1 s. Secondary structure of the proteins was assessed by visual inspection of CD spectra from 200 to 250 nm. The thermal denaturation or unfolding profile of the proteins was characterized by measuring the ellipticity changes at 222 nm induced by a temperature increase from 20 to 90°C with steps of 1 degree.

### Peptide synthesis

SMRT and GRIP1 peptides were synthesized using a CEM Liberty 1 Automated Microwave Peptide Synthesizer on a 0.05-*μ*mol scale, using solid-phase peptide synthesis (SPPS). For this technique, an N-protected C-terminal amino acid residue is anchored to an amino resin; in this case, H-Rink Amide Chem Matrix resin.

The amino acids were loaded onto this resin in a sequential manner from the C terminus to the N-terminus by repetitive cycles. Amino acids were Fmoc-protected and solubilized in dimethylformamide (DMF) to a concentration of 0.2 M. After every amino acid was loaded, they were deprotected using 20% piperidine in DMF to remove the Fmoc group. Following this, 0.5 M O-(1H-6-chlorobenzotriazole-1-yl)-1,1,3,3-tetramethyluronium hexafluorophosphate (HCTU) in DMF was used as an activator, with 3 M *N,N*-diisopropylethylamine (DIPEA) in NMP (N-methyl-2-pyrrolidone) as an activator base. The deprotection and amino acid coupling reactions were repeated in a linear manner for each amino acid to build the peptide sequence from the C terminus to the N terminus.

After synthesis of the full peptide sequences, the resin was removed and washed. The SMRT peptide was incubated with fluorescein isothiocyanate (FITC) in 5-fold molar excess for 5 h on a shaker at room temperature. The FITC-SMRT peptide and the GRIP1 peptide were cleaved from the resin by incubating the resin in 1 mL of TFA:TES:H_2_O at room temperature for 3 h. The peptides were precipitated using cold diethyl ether and then centrifuged at 3,500 × g for 5 min. Supernatants were discarded, and the pellets were washed twice more with diethyl ether. After the 3rd ether wash, the peptides were freeze-dried and left overnight before purification using semi-prep high-performance liquid chromatography (HPLC). The fractions containing peptide from HPLC were pooled and a sample was submitted for LC-MS to determine purity.

BODIPY-TMR C_5_ malemide (Invitrogen) was coupled to GRIP1 peptide through an N-terminal cysteine. Next, 90 *μ*M peptide was incubated with a 5-fold molar excess of BODIPY in a 1-mL reaction with constant stirring for 2 h in darkness at room temperature. The purification of the labeled peptide from free dye was performed using a PD-10 column (GE Healthcare) pre-equilibrated with 1 × PBS containing 0.5% Tris(2-carboxyethyl)phosphine hydrochloride. Eluted fractions were concentrated to 50 *μ*L using an Amicon centrifugal concentrator.

### Crystallisation, structure determination, and refinement

The P393GX LBD was mixed with a 5-fold molar excess of T3 (Sigma) and concentrated up to 9 mg/mL. Crystallization trials were initially conducted using commercial screens (Molecular Dimensions) into MRC 96-well sitting drop crystallization plates using 100 nL of protein sample and precipitant. Hexagonal crystals up to 20 *μ*m in length were grown via sitting drop vapor diffusion at room temperature using 0.2 M NaCl, 0.1 M Tris (pH 8.5), and 1 M lithium sulphate. Cryoprotectant solution containing 20% glycerol, in addition to the other crystallisation buffer components, was added to the crystals, which were then rapidly frozen at 100 K using liquid nitrogen. Data were collected at the microfocus beamline I-24 at the Diamond Light Source (UK). The structure was solved by molecular replacement using wild-type TR*α* LBD (PDB code 2h79) as a search model in Phaser. The preliminary model was rebuilt iteratively by multiple rounds of refinement and building using Refmac5 and Coot to an R_free_ of 22% and an R_work_ of 18%. The final model contains one molecule of P393GX and one molecule of T3 in the asymmetric unit. The final model has 95.73% residues in the favored region, 4.27% in the allowed regions, and none in the outlier region of the Ramachandran plot.

### Synthesis of TH analogues

**(i) Protection of T3 [tert-butyl (S)-2-((tert-butoxycarbonyl)amino)-3-(4-(4-hydroxy-3-iodophenoxy)-3,5-diiodophenyl)propanoate].** Perchloric acid (0.67 mL, 11.06 mmol, 6 eq) was added dropwise to a solution of T3 (1.20 g,1.84 mmol) in *tert*-butyl acetate(4.40 mL,0.033 mol, 18 equiv) at 0°C. Upon completion of the addition, the cooling bath was removed, and the reaction mixture was stirred for 1 h at room temperature. The reaction mixture was cooled to 210°C and excess acid was neutralized by the very slow dropwise addition of a solution of potassium carbonate (3.06 g, 0.022 mol, 12 equiv) in water (5.0 mL), keeping the temperature below 0°C. THF:water (1:1, 6.0 mL) and a solution of potassium carbonate (0.51 g, 3.69 mmol, 2 eq) in water (1.0 mL) were added followed by the slow dropwise addition of a solution of Boc anhydride (0.443 g, 2.03 mmol, 1.1 equiv) in THF (6.0 mL). The reaction mixture was warmed to room temperature and stirred for 2.5 h. The reaction mixture was extracted with EtOAc (3 × 30 mL) and the combined organic extract was dried over magnesium sulfate and then filtered and concentrated under reduced pressure to afford the crude product, which was purified by flash chromatography on silica (petroleum ether [40 to 60°C]: ethyl acetate, 3:1) to produce the protected T3 as a white solid (899 mg,1.11 mmol, 60%). MP: 85° to 86°C; FTIR (ATR/cm^−1^) *υ*_max_: 3355, 2968, 2932, 1703, 1688, 1577, 1508, 1441; ^1^HNMR (400Hz, CDCl_3_): *δ* 7.66 (s, 2H), 7.08 (d, J=3Hz, 1H), 6.89 (d, J=9Hz, 1H), 6.66 (dd, J=3, 9Hz, 1H), 5.15 (d, J =6Hz, 1H), 4.42 to 4.40 (m, 1H), 3.06 to 2.94 (m, 2H), 1.46 (s, 9H), 1.45 (s, 9H); ^13^CNMR (100Hz, CDCl3): *δ*170.4, 156.0, 153.0, 150.6, 150.3, 141.4, 138.0, 124.8, 117.4, 115.3, 90.8, 85.4, 83.1, 80.3, 54.8, 37.1, 28.2, 28.2. LRMS: (LC-MS) *m/z* calc. 806.91 [M+H]^+^, *m/z* found 805.8 [M–H]^–^.

**(ii) Protected ES08 {tert-butyl (S)-3-(4-(4-(([1,19-biphenyl]-4-ylsulfonyl)oxy)-3-iodophenoxy)-3,5-diiodophenyl)-2-((tert-butoxycarbonyl)amino)propanoate}**. To a stirred solution of protected T3 (75 mg, 0.09 mmol) in anhydrous dichloromethane (4.0 mL) were added (4-biphenylsulfonyl chloride [28 mg, 0.11 mmol, 1.2 eq], triethylamine [0.03 mL, 0.23 mmol, 2.5 eq], and 4-dimethylaminopyridine [0.57 mg, 0.005 mmol, 0.05 eq]) at room temperature under a nitrogen atmosphere. The resulting solution was stirred at room temperature for 16 h. The reaction mixture was quenched with water (15 mL) and the aqueous phase extracted with dichloromethane (3 × 30 mL). The organic layers were combined and washed with saturated sodium hydrogen carbonate solution (20 mL) and brine (20 mL). The organic layers were dried over magnesium sulfate and then filtered and concentrated under reduced pressure. Purification by flash column chromatography on silica (petroleum ether [40 to 60°C]: ethyl acetate, 5:1) afforded the title compound as a white solid (67 mg, 0.065 mmol, 71%). ^1^H NMR (400 Hz, CDCl_3_): *δ* 7.92 (d, J = 8 Hz, 2H), 7.69 (d, J = 8 Hz, 2H), 7.63 (s, 2H), 7.58 (d, J = 7 Hz, 2H), 7.47 to 7.40 (m, 3H), 7.23 (d, J=4Hz, 1H), 7.12 (d, J=3Hz, 1H), 6.69 (dd, J=3, 9Hz, 1H), 5.10 (d, J=6Hz, 1H), 4.37 (appd, J=5Hz, 1H), 3.03 to 2.90 (m, 2H), 1.41 (s, 9H), 1.41 (s, 9H); ^13^CNMR(100Hz, CDCl_3_): *δ*170.3, 167.5,154.7, 152.4, 147.6, 145.2, 141.5, 139.0, 138.5, 134.2, 129.6, 129.3, 129.0, 127.8, 127.6, 126.8, 123.7, 116.5, 90.4, 90.9, 83.1, 80.3, 54.8, 37.1, 28.2, 28.5.

**(iii) ES08 {(S)-3-(4-(4-(([1,19-biphenyl]-4-ylsulfonyl)oxy)-3-iodophenoxy)-3,5-diiodophenyl)-2-aminopropanoic acid}**. Protected ES08 (53 mg, 0.052 mmol) was introduced to a microwave vial and a mixture of water and acetic acid (ratio of 1:1, 0.52 mL) was added. The microwave vial was heated at 160°C for 20 min in a BIOTAGE microwave and then cooled to room temperature. The deprotection was monitored by liquid chromatography-mass spectrometry (LC-MS) analysis. The resultant mixture was transferred into a round bottom flask using a minimal volume of water. The mixture was concentrated under reduced pressure using a freeze dryer to remove residual water and acetic acid to produce ES08 as an off-white solid (18 mg, 0.021 mmol, 40%). MP: 209 to 212°C; FTIR (ATR/cm^21^); *υ*_max_: 3062, 2960, 2922, 1701, 1593, 1541, 1472; ^1^H NMR (400 Hz, DMSO): 8.01 (d, J = 8 Hz, 2H), 7.90 (d, J = 8 Hz, 2H), 7.83 (s, 2H, 2), 7.80 (d, J = 7 Hz, 2H), 7.57 to 7.47 (m, 3H), 7.21 (d, J = 9 Hz, 2H), 6.87 (dd, J = 2, 9 Hz, 1H), 3.12 (dd, J = 3, 14 Hz, 1H), 2.84 to 2.97 (m, 2H); LRMS: (LC-MS) m/z calc. 866.81 [M1H]^1^, m/z found 865.7 [M2H]^2^.

### Electrophoretic mobility shift and two-hybrid protein-protein interaction assays

Electrophoretic mobility shift assays using WT, E403X and E403K mutant TR*α* proteins and RXR*α*, and either canonical or human KLF9 promoter TREs (28) were undertaken as described previously (9).

For expression in transiently transfected mammalian cells, full-length WT and mutant TR*α* cDNAs were cloned in pCMX-VP16 (kind gift from R. Evans) to yield VP16-TR*α* fusions. Constructs which express the GAL4 DNA-binding domain (GAL4DBD) alone or are fused to receptor-interacting domains of the SMRT or NCoR corepressor isoforms and the TRAP220 coactivator have been previously described (29–31). The reporter gene (UASTKLUC) containing GAL4 binding sites, and the vector (Bos *β*-gal) used to correct for transfection efficiency, have also been previously described (32).

Human embryonic kidney cells (HEK293) seeded in medium (DMEM supplemented with 10% charcoal-stripped fetal bovine serum and 1% PSF (GIBCO-Invitrogen), were transfected using Lipofectamine 2000 (Invitrogen) with 16 ng of GAl4DBD-cofactor, 8 ng of VP16-TR*α*1, 80 ng UASTKLUC, and 8 ng Bos *β* gal expression vectors). Following exposure to fresh medium supplemented with either vehicle (DMSO), T3, or different thyroid hormone analogues, cells were harvested after 36 h and luciferase activity was normalized to Bos *β*gal activity. Results are shown as the mean +/− standard error of the mean (SEM) of at least 5 independent experiments, each performed in triplicate.

### Zebrafish studies

Zebrafish (Danio rerio) from wild-type (AB) and tg(kdrl:EGFP) adults were maintained in controlled conditions, and all procedures conformed to Italian law (D. Lgs no. 2014/26, implementation of the 2010/63/UE). WT and E303X mutant human THRA (ENST00000450525) vectors were linearized and transcribed in vitro using the mMESSAGE mMACHINET7 kit (Ambion), and RNA purified using the Megaclear Kit (Ambion). Zebrafish eggs (cell stage 1 to 2) were injected with hTR*α*1 transcripts (WT or E403X mutant) at doses of 80 pg per embryo. At 6 h postfertilization, the injected embryos were treated with 20 *μ*M DMSO (Sigma), 2 to 20 *μ*M triiodothyronine (IBSA-Farmaceutici), or 0.5 to 2 *μ*M ES08 added to the harvesting water. Morphological (cerebral edema, cardiac edema, altered body curvature, thinning of caudal vein plexus), vascular (incomplete formation, misplaced or aberrant branching of intersomitic vessels, and reduced area of caudal vein plexus) and skeletal (reduced or loss of mineralization of cleitrum and operculum, defective ceratohyal arch and ceratobranchial cartilages) anomalies were graded (0, normal; 1 to 4, increasingly abnormal) and used to compute an abnormal morphological index (AMI), vascular malformation index (VMI) or skeletal malformation index (SMI). Expression of known thyroid hormone responsive target genes in zebrafish (33, 34) was measured by quantitative real-time PCR as described previously (19).

### *Ex vivo* studies of patient-derived peripheral blood mononuclear cells and inducible pluripotent stem cells

Human inducible pluripotent stem cells (hIPSCs) were derived from primary, peripheral blood mononuclear cells of an RTH*α* patient with the E403X TR*α* mutation (9) by our NIHR BRC hIPSC core facility, as described previously (35). Following the exposure of cultured peripheral blood mononuclear cells or hiPSCs to T3 or analogue, the expression of KLF9, a thyroid hormone receptor target gene, was quantified as described previously (9).

### Statistical analysis

Comparisons of data were undertaken using unpaired, 2-tailed t tests. P values of less than 0.05 were considered significant.

## Figures and Tables

**Table 1:** Data collection and refinement statistics^a^

Data collection	P393GX: T3
Space group	P6_4_22
Cell dimensions	
a, b, c (Å)	143.33, 143.33, 88.50
*α*, *β*, *γ*(°)	90.00, 90.00, 120.00
Wavelength (Å)	0.9692
Resolution (Å)	71.06–3.00 (3.18–3.00)
*R_merge_*	0.130 (0.690)
I/*σ*I	22.0 (5.3)
Completeness (%)	100.0 (100.0)
Redundancy	16.5 (17.3)
Refinement	
Resolution (Å)	71.06–3.00 (3.18–3.00)
Total no. of reflections	185,125 (30,547)
R_work_/ R_free_	0.18/0.22
No. of atoms	
Protein	3824
Ligand	23
Water	9
B-factors	
Protein	60.78
Ligand	51.45
Water	40.95
RMS deviations^b^	
Bond lengths (Å)	0.0164
Bond angles (°)	2.0587

a Table indicates data collection and refinement statistics generated for the P393GX:T3 model. The number of crystals for this structure is one. Values in parentheses are for the highest-resolution shell.

b RMS, root mean square.

## Data Availability

The structure in Fig. 5D has been deposited with PDB ID 7QDT.
